# Changes in the Local Conformational States Caused by Simple Na^+^ and K^+^ Ions in Polyelectrolyte Simulations: Comparison of Seven Force Fields with and without NBFIX and ECC Corrections

**DOI:** 10.3390/polym14020252

**Published:** 2022-01-08

**Authors:** Natalia Lukasheva, Dmitry Tolmachev, Hector Martinez-Seara, Mikko Karttunen

**Affiliations:** 1 Institute of Macromolecular Compounds, Russian Academy of Sciences, Bolshoy Pr. 31, 199004 St. Petersburg, Russia; 2 Institute of Organic Chemistry and Biochemistry, Czech Academy of Sciences, Flemingovo Náměstí 542/2, CZ166 10 Prague 6, Czech Republic; hseara@gmail.com; 3 Department of Physics and Astronomy, The University of Western Ontario, 1151 Richmond Street, London, ON N6A 5B7, Canada; 4 The Centre of Advanced Materials and Biomaterials Research, The University of Western Ontario, 1151 Richmond Street, London, ON N6A 5B7, Canada; 5 Department of Chemistry, The University of Western Ontario, 1151 Richmond Street, London, ON N6A 5B7, Canada

**Keywords:** peptides and proteins, counterions, ions, carboxyls, molecular dynamics, force fields

## Abstract

Electrostatic interactions have a determining role in the conformational and dynamic behavior of polyelectrolyte molecules. In this study, anionic polyelectrolyte molecules, poly(glutamic acid) (PGA) and poly(aspartic acid) (PASA), in a water solution with the most commonly used K^+^ or Na^+^ counterions, were investigated using atomistic molecular dynamics (MD) simulations. We performed a comparison of seven popular force fields, namely AMBER99SB-ILDN, AMBER14SB, AMBER-FB15, CHARMM22*, CHARMM27, CHARMM36m and OPLS-AA/L, both with their native parameters and using two common corrections for overbinding of ions, the non-bonded fix (NBFIX), and electronic continuum corrections (ECC). These corrections were originally introduced to correct for the often-reported problem concerning the overbinding of ions to the charged groups of polyelectrolytes. In this work, a comparison of the simulation results with existing experimental data revealed several differences between the investigated force fields. The data from these simulations and comparisons with previous experimental data were then used to determine the limitations and strengths of these force fields in the context of the structural and dynamic properties of anionic polyamino acids. Physical properties, such as molecular sizes, local structure, and dynamics, were studied using two types of common counterions, namely potassium and sodium. The results show that, in some cases, both the macroion size and dynamics depend strongly on the models (parameters) for the counterions due to strong overbinding of the ions and charged side chain groups. The local structures and dynamics are more sensitive to dihedral angle parameterization, resulting in a preference for defined monomer conformations and the type of correction used. We also provide recommendations based on the results.

## 1. Introduction

Overbinding of charged moieties in classical MD simulations is a well-known problem that has its roots in force field parameterization [1,2,3,4,5]. The problem has been recognized for a long time but it persists and is important as simulations of soft matter and biological systems typically involve the presence of counterions, salt or both, and overbinding can have an influence on both their structural and dynamic properties. It can be rather severe, and several solutions have been proposed to resolve this issue. One of them is polarizable force fields [6,7,8], which allow for direct accounting of the polarization effects rendering the force field non-additive. However, there are only a limited number of molecules that have been parameterized for polarizable force fields, and parameterization is, in general, not straightforward. Polarizable force fields also require significantly more computational resources [9,10,11]. Other solutions involve the addition of corrections to electrostatic interactions into non-polarizable force fields [3,4,5,12]. Those simulations are computationally cheaper and currently cover a large number of biomolecules. There are two commonly used corrections, namely the ad hoc the non-bonded fix (NBFIX) [3] and the mean-field electronic continuum corrections (ECC) [13].

In NBFIX, the Lennard-Jones parameters between charged atoms are modified such that the contact distance between them is artificially increased, thus weakening their effective interactions. ECC reintroduced the missing electronic polarizability in non-polarizable force fields caused by the surrounding medium using a physically sound mean-field. This latter correction does not, however, make the force field polarizable (as partial charges are fixed). It has been shown that both of the corrections can alleviate the problem of artificial force field driven overbinding in many cases [5,14,15]. It is important to note that overbinding here refers to substantial increase in counterion binding due to uncertainties in force field parameterizations, while overcharging is a real physical phenomenon that occurs in various strongly charged systems including many biological systems (see for example [16,17]). Our previous study showed that NBFIX, although based on a heuristic scaling, works well with polyelectrolytes [14]. On the other hand, the ECC corrections, despite being based on a solid theoretical framework [4], do not always solve the problem [14]. It was shown using polyaspartic acid (PASA) and polyglutamic acid (PGA) that the outcome depends non-trivially on the underlying force field. In particular, the ECC correction did not solve the over-aggregation problem when applied directly to AMBER99SB-ILDN [18], leading, on occasion and counterintuitively, to even more enhanced overbinding [14].

**Figure 1:**
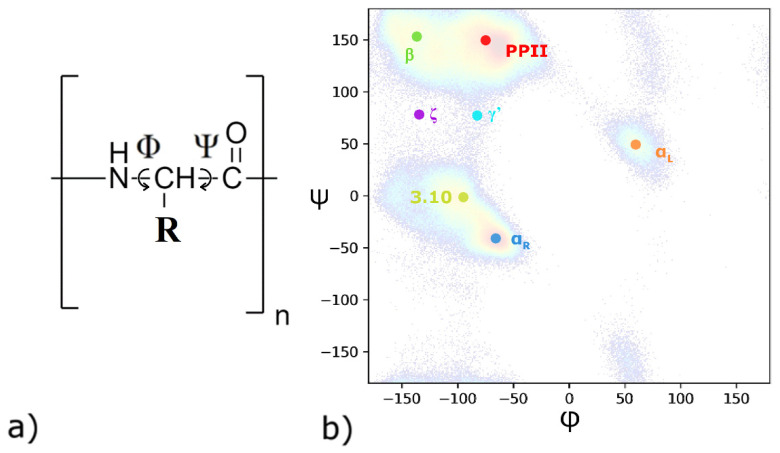
(**a**) Chemical structure of the PASA peptide. Rotations as defined by the torsion angles Φ and Ψ are indicated. (**b**) Ramachandran plot obtained using the CHARMM27 force field for PASA. Color code: green, 2.5_1_ helix (β-sheet); red, polyproline II helix (PPII); yellow, 3.10 helix; blue, right-handed α-helix (α_R_), orange, left-handed α-helix (α_L_); violet and cyan, ζ and γ’ conformations, respectively.

The sizes of the molecules are determined by their backbone conformations. Our previous study [14] demonstrated a strong dependence of molecular sizes (using PASA and PGA) on distinct force field parameters including those implemented in NBFIX and ECC charge-charge interaction corrections. Monomer conformational states are mainly determined by the sidechain monomer dihedral angles. Previously, Marchand et al. [19] demonstrated the strong influence of the counterion parameters on poly(glutamic acid) conformation in sodium chloride solution. However, the question concerning which force fields yield the most accurate result remains unanswered. One of the aims of the present study is to clarify the influence of the force field choice and counterion parameters on PASA and PGA monomer conformations. Simultaneously, we determine the predictive power of the tested force fields. This is conducted by comparing the results of the simulations with the existing experimental data. Instead, the global structure of a peptide is mostly fixed by the dihedral angles between the amino acid residues (Figure 1a). They can adopt energetically preferential conformational states which are usually presented as the most populated regions in classical Ramachandran (e.g., Φ, Ψ vs. population) plots [20,21]. The different nomenclatures of Ramachandran plots are well-described in the review by Hollingsworth and Karplus [21].

The populations of backbone conformations have been studied experimentally for aspartic and glutamic acid residues in proteins [22], short peptides [23,24], and polypeptides [25,26,27]. Jha et al. [22] analyzed the X-ray structures of 2020 chains longer than 20 residues. They determined the probability distributions of the three major backbone conformations (PPII, 2.5_1_ (β-sheet), and α_R_ helices) of all amino acid residues for the entire protein data base (PDB), with the exception of helices and sheets, as well as for intrinsically disordered proteins. Hagarman et al. [23] determined the distributions of the major backbone conformations in Gly-Glu-Gly peptides using NMR (nuclear magnetic resonance) and vibrational spectroscopy techniques. They selected glycine as a neighbor to minimize the nearest neighbor interactions so that the obtained propensities could be considered as intrinsic. Their results indicated that the conformational propensity of glutamic acid does not significantly depend on its protonation state. Grdadolnik et al. [24] used infrared and Raman spectra to determine the relative populations of the three major backbone conformations (PPII helix, β-sheet, and right-handed α-helix) in 19 amino acid dipeptides (including Asp and Glu both in ionized and non-ionized states). The major population was found to be either a PPII helix or a β-sheet for all dipeptides except Gly, whereas the α_R_ helix population was present only in small proportions (≤10%). The experimental CD (circular dichroism) and FTIR (Fourier-transform infrared spectroscopy) data for homo-polymers of Asp and Glu [25,26,27] evidenced a very low (close to zero) fraction of right-handed α-helices in the fully ionized state. Similar results have been obtained from UV (ultra violet) Raman spectra [28,29] for fully ionized PGA.

Experimental data concerning the effects of monovalent cation types exist only for PGA in NaCl and KCl solutions using CD and UV resonance Raman measurements [30]. The data show that Na^+^ and K^+^ cations have similar effects on the PGA molecule. No evidence of pair formation between Na^+^ and K^+^ cations and the side chain carboxylates and no specific dependence of the conformational structure on the cation type was reported.

In this study, we have tested PASA and PGA molecules using the same force fields (OPLS-AA/L, CHARMM27, CHARMM36m, CHARMM22*, AMBER99SB-ILDN) as in the previous study [14] with the addition of two recent force fields of the AMBER family: AMBER14SB and AMBER-FB15 with either Na^+^ or K^+^ counterions. This was carried out both with their native force field parameters and, when available, using corrections for electrostatic interactions (NBFIX [3] and ECC [31]). In our previous paper, we observed strong dependence on parameterization. However, the data could not be used to determine which parameterization is the most accurate [14]. The results of our present work using both structural static and dynamic properties together with a comparison to experimental data [22,23,24,25,26,27,28,29,30] allows us to provide some recommendations. These recommendations could be used as a guideline for a simulations of anionic poly(amino acids).

## 2. Model, Force Fields and Method

### 2.1. Model

**Figure 2:**
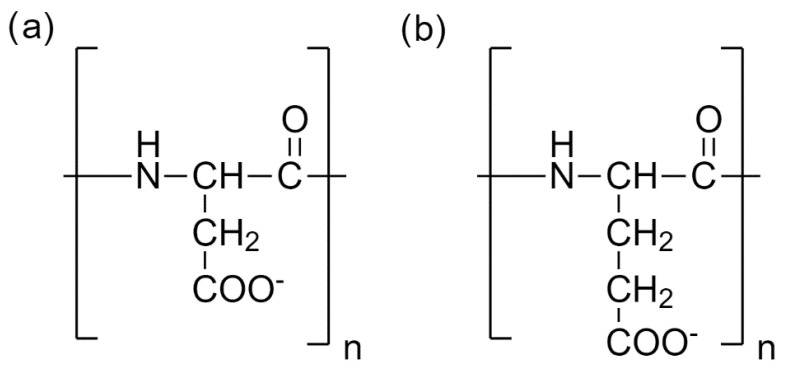
(**a**) PASA and (**b**) PGA chemical structures. The degree of polymerization is denoted by n. In our simulations, n = 32.

The model consisted of one fully deprotonated PASA or PGA molecule with 32 amino acids (Figure 2) in explicit water. All carboxyl groups were considered as fully deprotonated, which corresponds to pH > 7.4 [32]. Counterions were added to ensure overall charge neutrality. Two different counterions, Na^+^ and K^+^, were used. The simulation boxes were cubic with the linear size large enough (7 nm) to avoid self-interaction through periodic boundary conditions. The list of all the simulated systems is presented in Table S1. Research manuscripts reporting large datasets that are deposited in a publicly available database should specify where the data have been deposited and provide the relevant accession numbers. If the accession numbers have not yet been obtained at the time of submission, please state that they will be provided during review. They must be provided prior to publication.

### 2.2. Force Fields

We tested seven commonly used force fields together with their recommended ions and the NBIFX [3] and ECC [13] corrections. The force fields tested were: AMBER99SB-ILDN (FF99SB-ILDN) [19], AMBER14SB (FF14SB) [33], AMBER-FB15 (FF-FB15) [34], CHARMM27 (C27) (i.e., CHARMM 22/CMAP) [35], CHARMM36m (C36m) [36], CHARMM22* (C22*) [37], and OPLS-AA/L (OPLS) [38]. The water models were chosen according to the recommendation for each of the force fields, namely TIP3P [39] for all the force fields (except for OPLS, for which TIP4P [38] is the recommendation). For FF-FB15 we used the variant called TIP3P-FB [40] which is the recommendation [34]. For C36m we used TIP3P with non-zero van der Waals parameters for the hydrogen atoms as recommended [41].

### 2.3. ECC and NBFIX Corrections in Protein-Ion Interactions

We tested both NBFIX [3] and ECC [31] corrections. For NBFIX, the corrections were applied between the carboxyl groups (Figure 2) and counterions. NBFIX is included by default in the C36m force field for the interactions between the Na^+^ ions and the negatively charged carboxylic groups in glutamic and aspartic amino acids [36]. However, for K^+^ ions the NBFIX parameters are not included by default and in the current study they were taken from Yoo et al. [42]. We use the abbreviation C36m# NBFIX for CHARMM36m with NBFIX (for Na^+^ [included in the force field by default] and K^+^ ions [not included by default]). For the systems with CHARMM36m without NBFIX, we use the abbreviation C36m#. 

The ECC approach is based on scaling the atomic partial charges [42]. ECC has been successfully tested in our own [5,14,43] and other studies [44]. The original partial charges of the atoms in all carboxylic groups of PASA, PGA, including their C-termini and counterions (Na^+^ and K^+^ counterions), were scaled [43]. For the ions, the LJ σ-parameters for counterions are also scaled [45,46]. To indicate the systems with ECC corrections, we use the suffix ECC. Four force fields with ECC were considered: FF99SB-ILDN ECC, FF14SB ECC, FF-FB15 ECC, and C36m# ECC (without NBFIX corrections).

### 2.4. Method

All simulations were performed for 1 μs with 2 fs time step using either the GROMACS 2016.3 or 2018 package [47]. The first 100 ns was used for equilibration and the remaining 900 ns for data analysis. The isothermal-isobaric (NPT) ensemble at the temperature of 300 K and at 1 bar was applied using the Nosé-Hoover thermostat [48,49] and the Parrinello-Rahman barostat [50]. Long-range electrostatic interactions were treated using the particle-mesh Ewald (PME) method [51,52]. The lengths of the bonds with hydrogen atoms were constrained with the P-LINCS algorithm [53]. Visual molecular dynamics (VMD) software [54] was used for visual trajectory analyses. Errors were calculated as standard deviation.

## 3. Results

### 3.1. Ramachandran Maps and Backbone Conformations

To compare the results obtained in our MD simulations with the published experimental data, we have constructed Ramachandran maps for each tested system. First, we compared the forms of the distributions of the most populated Φ,Ψ regions with the possible conformational states known from the literature [21] (Figures S1 and S2). The Ramachandran plots for Asp and Glu residues presented in [21] show two densely populated areas of stretched (PPII and β-sheet) and screw (α_R_- and 3.10-helices) conformations, sparsely populated intermediate conformational regions (around ζ and γ’), as well as areas of α_L_ conformations.

Following the definitions from literature [55], we considered a residue to be in the α_R_ region of the Ramachandran map with angles −90° ≤ Φ ≤ −30° and −90° ≤ Ψ ≤ 0°, in the α_L_ region when 30° ≤ Φ ≤ 90°and 0° ≤ Ψ ≤ 90°, in the β-sheet region with angles of −180° ≤ Φ < −104° and 180° ≤ Ψ ≤ 104°, and in the PPII region with angles of −104° ≤ Φ ≤ −46°and 116° ≤ Ψ ≤ 174°. For 3.10 conformation it is known [56] that the average Φ, Ψ shifts systematically from approximately −58°, −32° to about −90°, −4° degrees. Regions centered at (Φ, Ψ) = (−80°, +80°), are called γ’, and at (−135°, 60°) are called ζ [21].

**Figure 3:**
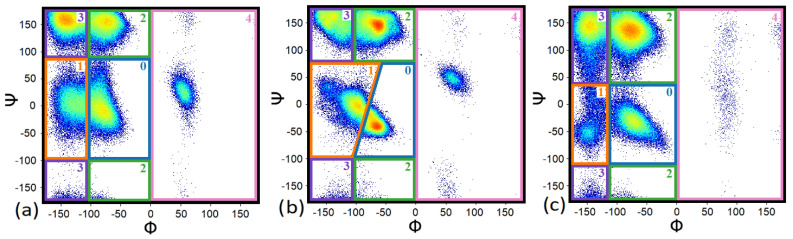
The integration regions of Ramachandran maps for PASA with Na+ using (**a**) FF99SB-ILDN, (**b**) C27 (also C22 and C36m), and (**c**) OPLS/AA force fields. The color boxes show the areas of dihedral angles related to the conformations: (0, α_R_ helix for C27 and also for FF99SB-ILDN and OPLS/AA), (1, 3.10 helix for C27 and additional screw conformation for FF99SB-ILDN and OPLS/AA), (2, PPII helix), (3, β-sheet), and (4, φ > 0°—mostly left handed α-helix).

Our data show that using FF99SB-ILDN, there are two main allowed conformational areas of the stretched and screw conformations and much less populated regions around ζ, γ’ and α_L_ (Figures S1 and S2). This holds for both PASA and PGA, and the shapes and positions of the areas of stretched conformations are in good agreement with the experimental data [21]. The stretched conformations (PPII and β-sheet) have the highest probability for the allowed conformational states. The areas of screw conformations are relevant but seemingly wider than the experimentally obtained ones [21] due to the addition of lesser populated areas around α_R_- and 3.10-conformations (e.g., Φ from −150° to −100° and for Ψ from −50° to 50°). These additional areas are not typical for aspartic and glutamic acid residues in peptides [21]. The conformations from these areas are referred to as “additional screw” conformations below. For FF99SB-ILDN, a single continuous region is observed in the area of the dihedral angles −100° ≤ Φ ≤ −50° and −100° ≤ Ψ ≤ 100° (Figure 3a) with a maximum which can be related to the α_R_-conformations [55]. Interestingly, the introduction of ECC leads to increased populations in this region.

For FF14SB the total area of accessible conformations is larger compared to FF99SB-ILDN (Figures S1 and S2). The emerging new screw conformational states ζ and γ’ [21] for both PASA and PGA form a common highly populated region extended below the stretched β-sheet and PPII conformations. The common area for screw α_R_- and 3.10-conformations are shifted to lower negative Ψ angles and are much narrower and lesser populated in comparison with FF99SB-ILDN. The region of the “additional screw” conformations similarly narrows and shifts down but becomes more populated. For FF14SB, the maximal populations remain in the top left quadrants of the Ramachandran maps (and in particular for β-sheet and ζ-conformations). The introduction of ECC corrections has only a minor effect on the Ramachandran plots.

For FF-FB15, the areas of stretched conformations for PASA (Figure S1) are slightly shifted to higher Ψ angles (Ψ~170) in comparison with FF99SB-ILDN (Ψ~165°), FF14SB (Ψ~150°), and experiments [21]. For PASA, the common region of the screw α_R_- and 3.10-conformations is difficult to separate from γ’. The γ’ region is sparsely populated in comparison with the ζ region. The β-sheet region has the highest population. For PGA, the maximal and comparable populations are for the β-sheet and PPII conformational regions. The stretch (PPII and β-sheet) and screw (α_R_- and 3.10) areas merge through the ζ-and γ’-conformational areas into one large heterogeneously populated region, with PPII and β-sheets having the highest populations. Overall, for both PASA and PGA, transitions between the stretch and screw conformational states are enhanced. This is the result of the parameterization of this force field, which allows for more significant thermodynamic fluctuations away from the local minima of the β-, PPII and α_R_-conformations [34]. Inclusion of ECC leads to an increasing population of the region common for α_R_- and 3.10-conformations for both PASA and PGA and ζ -conformational regions for PASA.

In terms of the shapes and positions of the maxima, the distributions for both PASA and PGA obtained with C27 and C36m (Figures S1 and S2) are the closest to the experimental ones [20]. These force fields, due to direct use of CMAPs for the peptide backbone, were designed to produce the correct (in relation to QM and X-ray data) dihedral angle distribution [40,41]. For these force fields, the areas of the stretched (PPII and β-sheet) and screw (α_R_ and 3.10) conformations are narrow and clearly separated. For C36m, the area of screw conformations is extremely narrow for Ψ in comparison with all other force fields. For C27 and C36m, each allowed area has only one conformation with high probability. In the case of C27, both conformations α_R_ and PPII have comparable probabilities, but for C36m the probability of PPII is much higher. The introduction of NBFIX and ECC to C36m# does not lead to any significant changes in the Ramachandran maps. The intermediate zones between the allowed areas for C36m (both with and without corrections) are very sparsely populated in comparison with the AMBER force fields.

For C22, three densely populated areas (around β, PPII and α_R_ + 3.10) are observed (Figures S1 and S2) with higher probability for PPII in comparison with α_R_ + 3.10 and β-sheets. The increased population in the γ’-conformational area (as compared to C27 and C36) is observed without the formation of a well–defined region as was the case for FF14SB and FF-FB15. The distributions for OPLS-AA also differ from the experimental data [21]. In the case of OPLS-AA, there are two allowed conformational areas with high probabilities of PPII, β-sheets and α_R_-helices, and an increased population in the intermediate regions between stretched and screw conformations as compared to CHARMM force fields, but without the formation of well-defined allowed conformational areas as for AMBER force fields. This holds for both PGA and PASA.

In the experimental data for aspartic acid residues [21], while the area of the α_L_-helical conformations is highly populated, for glutamic acid it is only sparsely so. For C27, FF99SB-ILDN and FF14SB, this region is densely populated and well-defined not only for PASA but for PGA also. The result for PGA contradicts the experimental data [21]. The addition of ECC to FF99SB-ILDN and FF14SB increases the α_L_-helical population. For FF-FB15, the population of α_L_-helical conformations increases for both PASA and PGA (only with Na^+^ counterions). With the addition of ECC it increases even more. For C36m (with and without corrections), the α_L_-helical population increases only for PGA with both counterions. In all other cases the α_L_ helix conformational areas are low populated.

### 3.2. Fractions of Major Backbone Conformations

To better quantify the different conformational states, their fractions were calculated. The Ramachandran plots were divided into five regions. The fraction of each conformational state was identified as the integral of this specific area of the distribution. Since the maps obtained with different force fields are not similar, the areas for integration were chosen differently for the different force field families (see Figure 3).

For FF99SB-ILDN and OPLS/AA (Figure 3a,c), the areas indicated by number 0 are the regions that are common for α_R_- and 3.10-conformations and which cannot be separated each from other. The values of the dihedral angles of the maxima of this region can be related to the α_R_- region (−90° ≤ Φ ≤ −30° and −90° ≤ Ψ ≤ 0°, as it is generally specified [55] and was mentioned in the previous part). Taking this into account, the integration results over the area labeled 0 are considered as the fraction of the α_R_-conformations. The areas labelled 1 are the regions of the “additional screw” conformations as discussed in the previous section. Opposite to FF99SB-ILDN and OPLS/AA, for C27 (Figure 3b) the α_R_- and 3.10-conformations can be separated and the integration results over the areas labeled 0 and 1 are considered as the fractions of the α_R_- and 3.10-conformations, respectively.

The areas of integration for FF14SB and FF-FB15 differ from the FF99SB-ILDN areas (Figure S3) as well as each from other. For FF14SB, and for both PASA and PGA, the areas labelled 3 are the regions of the β-sheet and ζ-conformations do not separate and the integration results are considered entirely as the fraction of β-sheet conformations. In addition, integration over the areas labelled 2 that include PPII and γ’-conformations are reported as the fractions of PPII conformations. The integration results for areas labelled 0 give the fractions of the α_R_-conformations and the integration results for the areas 1 give the fraction of the “additional” screw conformations. For FF-FB15, due to their continuity the regions of γ’- and ζ—conformations were included in areas 0 and 1, respectively, and the integration results are considered as the fractions of the α_R_-conformations and the “additional screw” conformations. The areas of integration for C22 and C36m are equal to those for C27.

**Figure 4:**
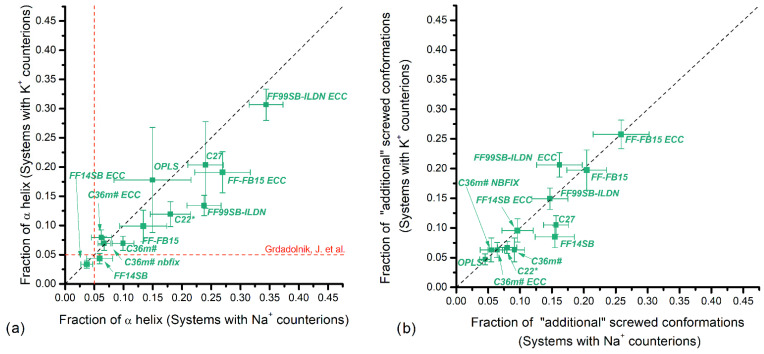
PASA: Fractions of (**a**) α_R_-helical and (**b**) 3.10-helical (for CHARMM force fields) and the “additional screw” conformations (for AMBER and OPLS force fields) with Na^+^ vs. K^+^ counterions. Vertical and horizontal dashed lines correspond to available experiment data.

**Figure 5:**
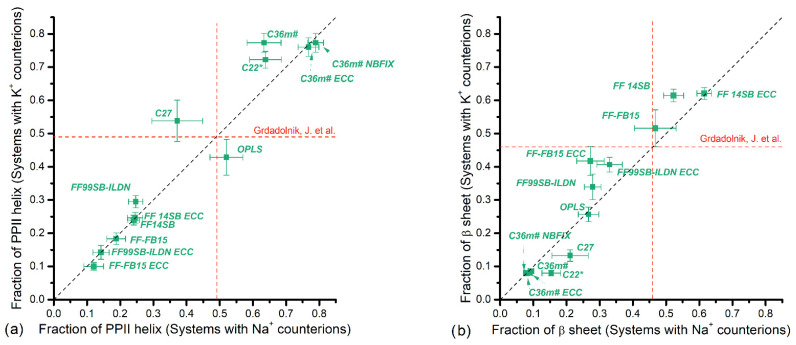
PASA: Fractions of (**a**) PPII helices and (**b**) β-sheet conformations with Na^+^ vs. K^+^ counterions. Vertical and horizontal dashed lines correspond to available experiment data.

**Figure 6:**
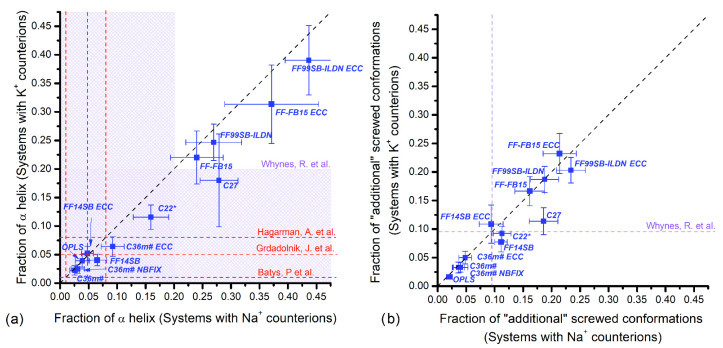
PGA: Fractions of (**a**) α_R_-helical and (**b**) 3.10-helical conformations (for CHARMM force fields) and the “additional screw” conformations (for AMBER and OPLS force fields) with Na^+^ counterions versus K^+^ counterions. Vertical and horizontal dashed lines and shadowed areas correspond to available experiment data.

**Figure 7:**
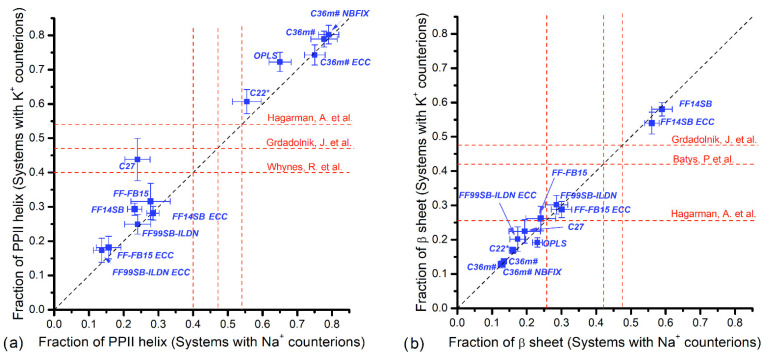
PGA: Fractions of (**a**) PPII helices and (**b**) β-sheet conformations with Na^+^ vs. K^+^ counterions. Vertical and horizontal dashed lines correspond to available experiment data.

The results including the effect of Na^+^ and K^+^ counterions are presented in Figure 4, Figure 5, Figure 6 and Figure 7. The horizontal axis represents fractions in systems with Na^+^ counterions, and the vertical axis with K^+^. The dotted diagonal corresponds to the results that are independent of the counterion type; this independence is suggested by experimental observations for PGA in NaCl and KCl solutions [30]. We are not aware of similar experiments using PASA but similar behavior is assumed. Table 1 lists experimental data regarding populations of the major backbone conformations for aspartic (ASA) and glutamic (GA) acid residues. These data are shown as vertical and horizontal dotted lines in Figure 4, Figure 5, Figure 6 and Figure 7.

#### 3.2.1. Poly (Aspartic Acid)

Figure 4 shows the fractions of the conformations corresponding to the α_R_- (Figure 4a) and 3.10-helical structures together with the “additional screw” conformations (Figure 4b) for PASA.

For the CHARMM family, the results for C36m including the ECC variant show the closest agreement with the experimental data from ionized dipeptides (5%) [24] and are close to the diagonal. This is explained by the improved parameterization that aims to decrease the α_R_-helix fraction compared to the previous versions of the CHARMM force fields; the fractions obtained with C22* and C27 have significantly higher values with C27 overstabilizing helical structures.

For the AMBER family, only FF14SB (with and without ECC) gives the α_R_-helical fraction close to the experimental one and is close to the diagonal. This is most likely due to the improvements introduced in FF99SB regarding the side chain rotational profiles for dipeptides with explicit α- and β-backbone conformations [33]. In contrast, FF-FB15 and FF99SB-ILDN (both with and without ECC) give significantly higher values for the α_R_-helix fraction with FF99SB-ILDN having higher values. The origin of this is most likely due to a better agreement of the amino acid conformational surfaces with the quantum chemical ones [34]. With the addition of ECC corrections, the fraction of α_R_-helices increases for both FF99SB-ILDN and FF-FB15. This may be explained by the weakening of the electrostatic repulsion between the charged carboxyl groups when ECC is added. The largest fraction of α-helical conformations >30% was obtained for FF99SB-ILDN ECC.

The results for OPLS, C27, C22*, FF99SB-ILDN, FF99SB-ILDN ECC, FF-FB15 and FF-FB15 ECC demonstrate some ion type sensitivity. For OPLS, C27, FF-FB15, and FF99SB-ILDN ECC this influence is small and within margin of error from the experimental data [24]. FF99SB-ILDN shows the largest sensitivity.

To the best of our knowledge, there is no experimental data for PASA regarding 3.10-helical structures. Among the CHARMM force fields, the lowest values for 3.10-helical structures were obtained for C36m (with and without corrections) and C22*. For the “additional screw” conformations, the largest fraction of was obtained for FF-FB15 ECC. Small/moderate dependence on counterion type was observed for C27, FF14SB, and FF99SB-ILDN ECC.

The fractions of the more stretched PPII-helix and β-sheet conformations for PASA are presented in Figure 5. As might be expected, the force fields giving the lowest values of the α_R_- and 3.10-helical fractions give the largest values for the stretched conformations. This is true for the sum of the PPII and β-conformations, but their relative contributions differ strongly for the different force fields. The CHARMM family, especially C36m (in all cases) and C22*, give extremely high fractions of PPII and extremely low β-sheet fractions. Similar tendency was observed for C27, which gave intermediate PPII fractions and relatively low value for β-sheets. The AMBER family, however, demonstrates the opposite tendency (i.e., low PPII fractions and relatively high β-sheet fractions). Experimental data [24] for the charged aspartic acid (ASA) dipeptide shows fractions comparable to each other, 49% and 46%, for PPII and β, respectively. None of the force fields is in agreement with experimental data for dipeptide conformations [24] (Table S2).

ECC corrections with FF99SB-ILDN and FF-FB15 lead to an increase in the screw (α_R_; 3.10 and “additional”) conformations and, consequently, to a decrease in the stretched (PPII and β) conformations for PASA. For FF14SB the effect of ECC is opposite. For PASA with K^+^, adding ECC has only a minimal effect. C36m showed only minor effects with both corrections. Overall, this makes ECC less predictable when applied to the AMBER force field family.

The results show that for PASA, the older CHARMM force fields (C27 and C22*) overestimate the fraction of the α-helical conformations. For the AMBER family, overestimation of the α-helix fraction is not only observed for the older FF99SB-ILDN but also the newest FF-FB15. In addition, the ECC correction appears unpredictable and usually worsens the situation when applied to the AMBER force field family. This is not surprising considering that AMBER force fields have highly delocalized charges effectively mimicking charge scaling [17].

Summarizing, none of the force field can be fully recommended as the most suitable for the PASA simulation when the dipeptide experimental data is taken into consideration (the numerical results for PASA are available in Table S2).

#### 3.2.2. Poly (Glutamic Acid)

Similar to PASA, the lowest fractions of the α_R_-helical conformations for PGA were obtained for C36m and FF14SB, Figure 6a. The fractions of the α_R_-helical conformations lying in the interval of the experimental values ~5–10% (8% for GA, 5% for di-GA, and <20% for PGA; see Table 1) were obtained for C36m# ECC and FF14SB with ECC. The original FF14SB, OPLS and the rest of the C36m flavours are in the slightly lower 2–5% interval [29]. Slightly above the experimental expected range, we find C22* and C27 (with K^+^). With the exception of C22* and C27, no significant dependence on counterion type was observed.

For FF-FB15 and FF99SB-ILDN (both with and without ECC) the α_R_-helix fractions exceed 20% with FF-FB15 yielding lower values of the two. The ECC corrections lead to an increase in the fraction of α_R_-helices due to excessive weakening of the electrostatic repulsion between the charged carboxyl groups [17]. As in the case of PASA, the largest fraction of α_R_-helical conformations was obtained for FF99SB-ILDN-ECC (~40%). Most force field variants show negligible or small dependence on the counterion types and only C22*, C27, FF99SB-ILDN, and FF-FB15-ECC show significant changes on the α_R_-helical conformations with different counterions, C27 showing the largest changes.

The fraction of the 3.10-conformations was estimated experimentally in Ref. [29] to be 9% (Table 1). In Figure 6b, we show the simulated values and their counterion dependence. The estimation of the 3.10-conformations for C36m (with and without corrections) gives ~3–5% for both counterions, and for C27 with K^+^ gives (within the margin of error) values near the experimental 9%. C27 is the only force field that showed a difference (albeit very small) between the Na^+^ and K^+^ counterions with PGA. For the other force fields, the 3.10-conformations were not present. Instead, “additional screw” conformations were found in regions not populated in the experimental Ramachandran plot for PGA. Their fractions demonstrate great deviations (with OPLS having the lowest values).

The fractions of the stretched conformations for PGA with K^+^ and Na^+^ counterions are presented in Figure 7a (PPII) and Figure 7b (β-sheets). Experimental values [23,24,29] for PPII lie in the interval 0.40–0.54 (Table 1). C22* yields consistent values with both K^+^ and Na^+^, while C27 agrees only for K^+^. Very large values were obtained for OPLS and C36m (with and without corrections), and all AMBER family force fields gave low values. OPLS, C36m and Amber family showed no significant dependence on counterion type.

Experimental values [23,24] for β-sheet fractions are 26% (for GA—glutamic acid [22]), 48% (for di-GA—dimer of glutamic acid [28]) and 42% (for PGA [57]) (Table 1). The values obtained using C27, FF99SB-ILDN and FF-FB15 (with and without ECC) for both counterions are close to 26% [22] (Figure 7b and Table S3). In all other cases, the values are above 50% (FF-14SB with and without ECC) or below 20% (FF99SB-ILDN ECC, C22* and C36m with and without corrections). No dependence on counterion type was observed. Numerical data for the PGA conformational fractions are collected in Table S3.

In summary, no single force field is in agreement with the available experimental data. FF99SB-ILDN and FF-FB15 drastically overestimate the α_R_-helical conformations. This is even worse with ECC. C22*, C27, and OPLS also overestimate screw conformations. Only C36m force fields and its variants produce compatible results on screw conformations. Unfortunately, these better-balanced force fields for the screw/stretch ratio fail to provide correct ratios between the PII and β structures. Only C27 for PGA with K^+^ counterions gives the α_R_-, 3.10, PPII and β fractions in agreement with the experimental data. Due to this agreement, C27 could be recommended for PGA simulations but limited to K^+^ counterions.

### 3.3. Lengths of the Conformational Sequences in PASA and PGA

**Figure 8:**
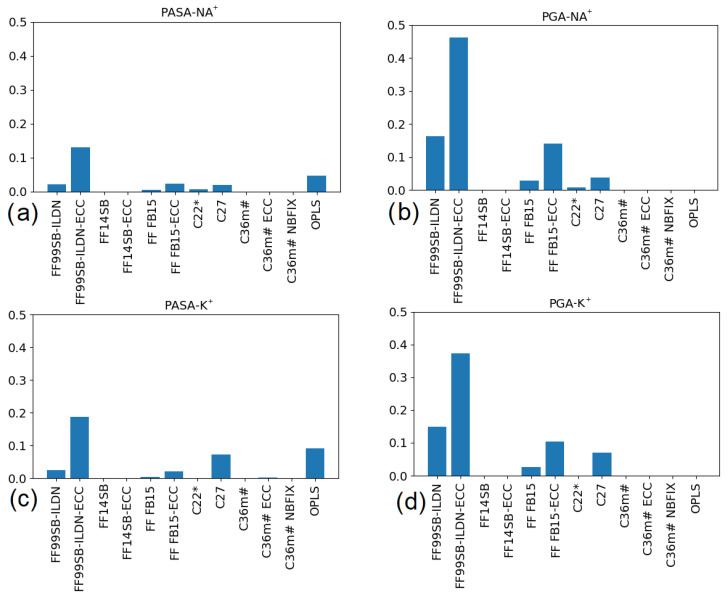
Fractions of PASA (**a**,**c**) and PGA (**b**,**d**) monomers included in the α_R_-helical sequences (more than three monomers).

This above estimation of the conformational fractions does not provide information about the distribution of a given conformer in a sequence. Circular dichroism (CD) allows the evaluation of secondary structure, including α_R_-helical sequences. For PASA and PGA, CD has demonstrated the absence of helicity for the fully ionized states of these two molecules [25,26,27]. Considering that an α_R_-helix has 3.6 residues per turn, only regions that form at least one helical turn (more than three monomers) can be part of a real α_R_-helix structure. Therefore, we can estimate the fractions of monomers constituting α_R_-helical sequences using the equation:

(1) $$F\alpha =\frac{\sum_{i,j}\frac{i}{30}R_{ij}}{t},$$

where *i* is the target number of contiguous monomers with α_R_-helical compatible backbone dihedrals in a peptide (from 4 to 30), *R_ij_* is the number of regions identified with length *i* in frame j in the simulation trajectory, and *t* is the number of the analyzed frames. The number of analyzed monomers per peptide was 30 (the chain length minus terminal monomers). First, we calculate the number of regions with a given length *i* of neighbors in the same conformational state. Figures S4 and S5 show the distributions of lengths for each system. Then, Equation (1) was used to calculate the fractions of monomers in α_R_-helical sequences as presented in Figure 8. The values can be compared with the α_R_-helical states obtained from the Ramachandran maps (Figure 4a and Figure 6a).

For PASA, comparison of the data in Figure 4a and Figure 8a,c shows that for FF99SB-ILDN-ECC (with Na^+^ and K^+^), C27 (with K^+^) and OPLS (with K^+^) nearly half of the monomers in the α_R_-helical conformations are included in the equiconformational sequences with lengths more than three monomers. For the force fields, the fractions of the α_R_-helical monomers in equiconformational sequences are much smaller (i.e., most of the monomers in α_R_-helical conformations are not in these sequences).

For PGA, the data in Figure 6a and Figure 8b,d show that the fractions of monomers in α_R_-helical conformations, and those included in sequences, are very close to each other in the case of FF99SB-ILDN-ECC (with K^+^ and Na^+^). This implies that practically all monomers having α_R_-helical conformations can be included in equiconformational sequences. For FF99SB-ILDN (with K^+^ and Na^+^) and C27 (with K^+^) nearly half of the monomers in α_R_-helical conformations form α_R_-helical conformational sequences. For C27 (with K^+^) as leading the candidate for PGA modeling with K^+^ counterions, this property needs to be taken into account. For FF-FB15-ECC (with K^+^ and Na^+^) about 30% of the α_R_-helical monomers are included in the sequences with the same monomer conformations. In all other cases the fraction is much lower.

### 3.4. Correlations of Molecular Sizes (R_g_) with Fractions of Stretched Monomer Conformations

**Figure 9:**
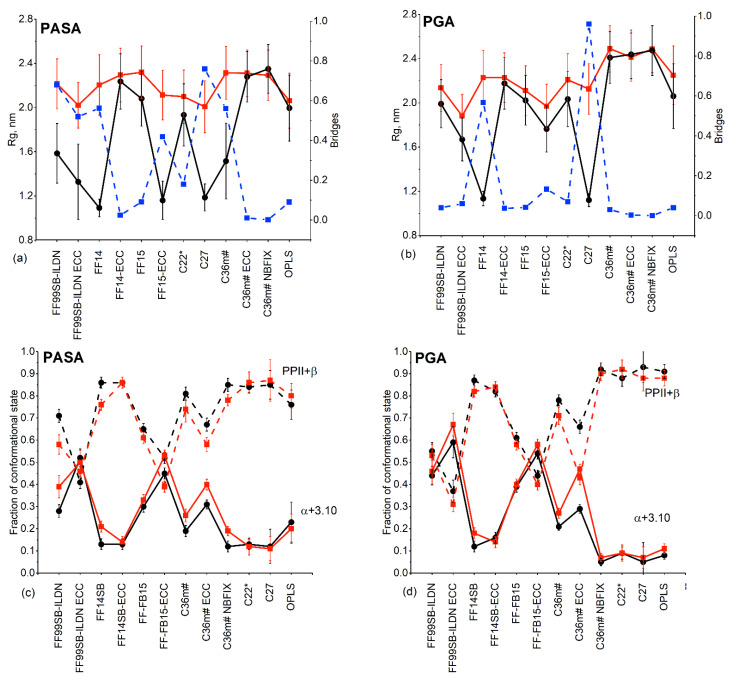
R_g_ (solid lines) and the fraction of carboxyl groups involved in the Na^+^ bridges formation (dashed lines) for (**a**) PASA and (**b**) PGA. The fractions of the stretched (solid lines) and screw (dashed lines) conformations for (**c**) PASA and (**d**) PGA. Black lines indicate the systems with Na^+^, red lines the systems with K^+^. Blue lines show fractions of carboxyl groups connected by Na^+^ bridges.

Figure 9 shows the radii of gyration (R_g_) and the fractions of the stretched and screw conformations. The stretched conformations are sums of PPII- and β-conformations, and the screw conformations are the sums of the α_R_- and 3.10- conformations.

R_g_ showed counterion dependence for PASA and PGA (Figure 9a,b). For PASA, the strongest dependence was observed for FF99SB-ILDN (with and without ECC), FF14SB, FB15-ECC, C27 and C36m#. In all these cases, the R_g_ values are smaller with Na^+^ counterions. For PGA, strong discrepancy on the counterion type was observed for FF14SB and C27. Formation of long-lived Na^+^ bridges between distant carboxyl groups, as was shown in our previous work [14], results in smaller R_g_ values. Fractions of carboxyl groups connected by Na^+^ bridges were calculated according to the procedure described in SI and are presented in Figure 9a,b using blue dashed lines. Considering that experimental results suggest no differences between Na^+^ and K^+^ [30], we conclude that such interactions may be an artifact. Therefore, force fields promoting such interactions are likely not correct.

Sensitivity of the molecular sizes to backbone dihedral angle parameterization is most pronounced with Na^+^ counterions when using AMBER force fields with and without ECC corrections. For FF99SB-ILDN and FF-FB15, the introduction of ECC corrections results in a decrease in the molecular sizes due to stronger stabilization of screw conformations as result of a counterintuitive and undesirable increase in Na^+^ bridges between the carboxyl groups. The native FF99SB-ILDN and FF-FB15 have high propensity of screw (α_R_-helix) conformations [58,59]. For charged molecules, electrostatic repulsion acting along the chain leads to chain stretching. ECC weakens the repulsion between the carbonyls which surpasses the reduction in Na^+^-carbonyl interaction and changes the solvation of the two groups in a way that leads to an increase in the α_R_-helical content with the corresponding decrease in R_g_. The opposite is observed for FF14SB and C36m (for PASA) which display the expected behavior. Compared to FF99SB, the native FF14SB has reparametrized side chain dihedral angles for better reproduction of sequence-dependent secondary structures (with α- and β-backbone conformations) [33].

**Figure 10:**
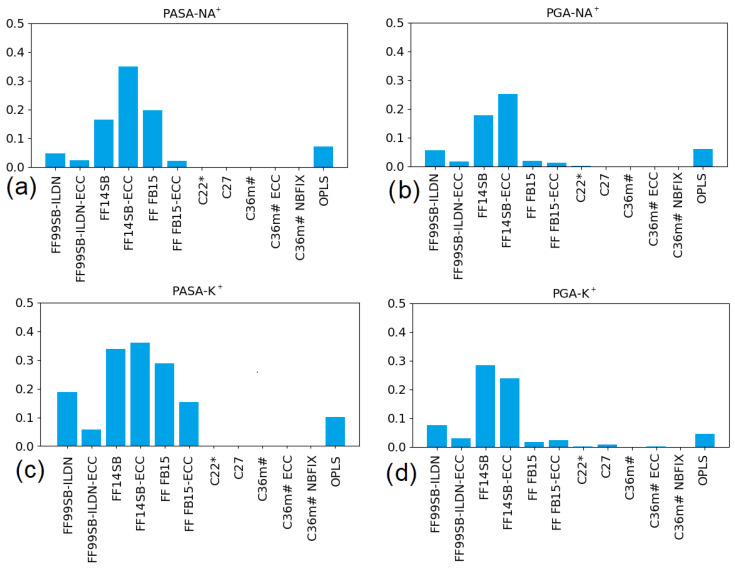
Fractions of PASA (**a**,**c**) and PGA (**b**,**d**) monomers included in the β-conformational sequences (more than 3 monomers).

FF14SB gave high fractions of β-backbone conformations (Tables S2 and S3) exceeding experimental values [22,23] and, moreover, β-conformational sequences (calculated according (1)) were formed (see Figure 10). Comparing the values in Figure 5b, Figure 7b and Figure 10, it can be concluded that about 30% of monomers with β-conformation in PASA and PGA with Na^+^ form β-conformational sequences. In contrast, with K^+^ about 50% of the β monomers are in β- conformational sequences and the occurrence of longer (≥5) sequences is higher than with Na^+^ (Figures S4 and S5). β-conformational sequences, in turn, can form β-sheets in which such sequences are linked by hydrogen bonds. For deprotonated PASA and PGA this role could be potentially played by counterions. For monovalent counterions we suspect this is an artifact.

**Figure 11:**
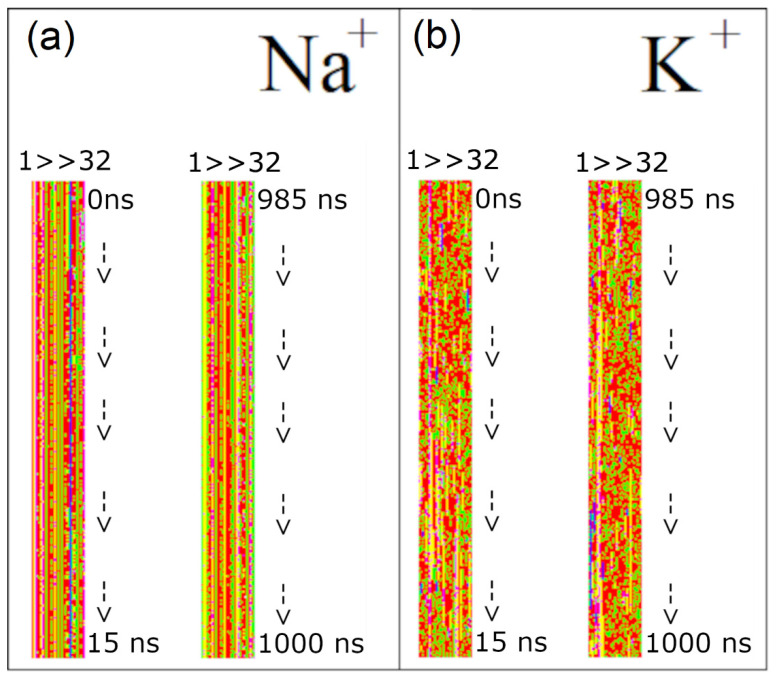
Change of dihedral angles of PASA’s main chain in each monomer in a simulation using FF14SB. Typical sections (at the start of the simulation (0–15 ns), at the end of the simulation (985–1000 ns)) (**a**) for a system with Na^+^ counterions and (**b**) for a system with K^+^ counterions. Each pixel illustrates the conformation of one monomer during 100 ps. The colors indicate the combination of φ and ψ dihedral angles corresponding to specific secondary structures: PPII helix, green; β-sheet structures, red; 3.10 helix, yellow; right-handed α helix, pink; left-handed α helix, blue.

For both PASA and PGA with Na^+^, the fraction of monomers in the β-conformational sequence is approximately 30% smaller than with K^+^ when using FF14SB (Figure 10). This is due to formation of β-sheets that is promoted by the Na^+^ bridges connecting the carboxyl groups of antiparallel short β-conformational sequences. This is illustrated in Figure 11 by the time evolution of PASA monomer conformations. For PASA with K^+^, there is a large number of β-sequences appearing and disappearing at different locations whereas with Na^+^ there are long-lived pairs of short β-sequences. Adding ECC corrections (FF14SB-ECC) weakens the electrostatic attraction between the Na^+^ ions and the carboxyl groups. As a consequence, β-sheet structures become disrupted and the fractions of monomers in β-sequences increase close to the expected values (that is, to the same ones as with K^+^). For this reason, R_g_ increases.

In contrast to R_g_, for which pronounced counterion-induced differences due to ion bridge formation were observed, the local sensitivity to the type of counterion (for the fractions of stretched (or screw) monomer conformations, Figure 9c,d) is less significant for all force fields. The same holds for α_R_-, 3.10 or additional screw, PPII and β-conformations (Figure S6). Correlation between R_g_ and fractions of stretched conformations of PASA and PGA monomers was observed in most cases. An increased (decreased) value of R_g_ is the result of an increased (decreased) fraction of stretched conformations. The opposite behavior was found for PASA and PGA using FF14SB (see the explanation above).

In summary: (1) an inverse relation for fractions of carboxyl groups connected by Na^+^ bridges and R_g_ was observed; (2) introduction of the ECC corrections to AMBER force fields leads to similar effects gives for two of them (FF99SB-ILDN and FF-FB15) and to the opposite for FF14SB (R_g_ increases). This is due to differences in their parameterization of the backbone dihedral angle potentials; (3) the lengths of the molecular fragments with similar conformational structures differ between force fields. When Na^+^ bridges are prominent, these fragments are long-lasting; and (4) the fraction of stretched/screw conformations is mostly counterion independent.

We calculated end-to-end and dihedral angle autocorrelation functions and determined the relaxation times by with exponential functions,

(2) $$f\left(t\right)=A\left(exp{\left(\frac{-t}{\tau }\right)}^{\beta }\right),$$

where *β* is the stretching exponent defining the width of the relaxation time distribution, *τ* is the average relaxation time and *t* is the simulation time. The amplitude *A* defines the contribution of the exponential function to the autocorrelation function.

**Figure 12:**
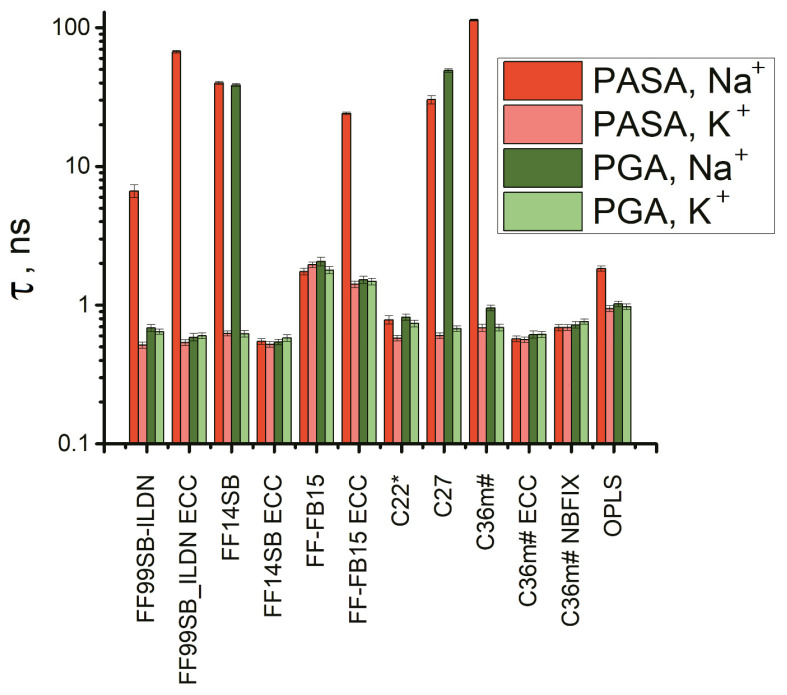
Relaxation times of the end-to-end distance in PASA and PGA molecules with K^+^ and Na^+^ counterions. Computed using Equation (2).

The end-to-end distance autocorrelation functions are presented in Figure S7 and the corresponding relaxation times in Figure 12. Six force fields stand out: FF99SB-ILDN (with and without ECC), FF14SB, FB15-ECC, C27, and C36m#. All of them with Na^+^ show strongly decreased chain dimensions (Figure 9) and display the relaxation times that are one to two orders of the magnitude larger compared using K^+^. For PASA, this was observed with all of the above six force fields. With PGA, this occurred for FF14SB and C27. Thus, long end-to-end relaxation times are specific for the systems with Na^+^ bridges (see Figure 9a,b).

Next, the autocorrelation functions for the Φ, Ψ dihedral angles were computed (Figure S8). In the following we focus only on the Ψ angle since the Φ angle motion is very limited (see SI). The Ψ angle autocorrelation functions in Figure S8 and the corresponding relaxation times in Figure 11 show dependence on both the force field and counterion type. Since the sampling step in the simulation trajectories was 0.1 ns, and the correlation times of K^+^ with FF99SB ILDN are shorter, only relaxation times longer than 0.1 ns are shown.

Strong counterion specificity was observed for C27 and FF14SB. Unrealistically strong interactions between the Na^+^ ions and the carboxyl groups in the PASA and PGA molecules led to the formation of ion bridges and a consequent increase in the relaxation time in comparison with K^+^ for C27. In the case of FF14SB, the increased relaxation time for Na^+^ in comparison with K^+^ is due to the formation of β-sheets (see the previous section) with β sequences connected through COO- groups by Na^+^ bridges. With the ECC corrections, the weakening of the electrostatic attraction between the Na^+^ ions and the carboxyl groups leads to β-sheets often becoming disrupted and as a result, the relaxation times usually decrease. The introduction of the ECC corrections to C36m# also leads to a decrease in the relaxation times of two to four orders of magnitude. For PASA with Na^+^ this can be explained by the destruction of the Na^+^ bridges due to the weakening of the electrostatic attraction. For PASA with K^+^, the decrease of τ is due to the weakening of the electrostatic repulsion between the side chain carboxyl groups and related decrease of the barriers inhibiting internal rotation of the main chain of PASA. This is the same is for PGA with Na^+^ and K^+^. That the behavior of PGA with Na^+^ is the similar as with K^+^ is explained by the fact that the Na^+^ ion bridges do not form in PGA (see Figure 9, and Ref. [14]) due to the entropic losses for longer side chains.

For the force fields for which there were no ion bridges, counterion specificity of the relaxation time was either practically absent (FF-FB15) or, as in the cases of C22 for PASA and OPLS, and C36m# for PGA, the relaxation times with K^+^ were higher than with Na^+^. The origin of the opposite effect is due to a different impact of the counterions on electrostatic repulsion of carboxyl groups. K^+^ counterions have only small influence on these groups’ repulsion that leads to an increased probability of extended conformations (PPII for these force fields are shown in Figures S1 and S2). On the other hand, interactions of Na^+^ ions with the carboxyl groups are much stronger and shield their electrostatic repulsion and result in a lower transition barrier.

**Figure 13:**
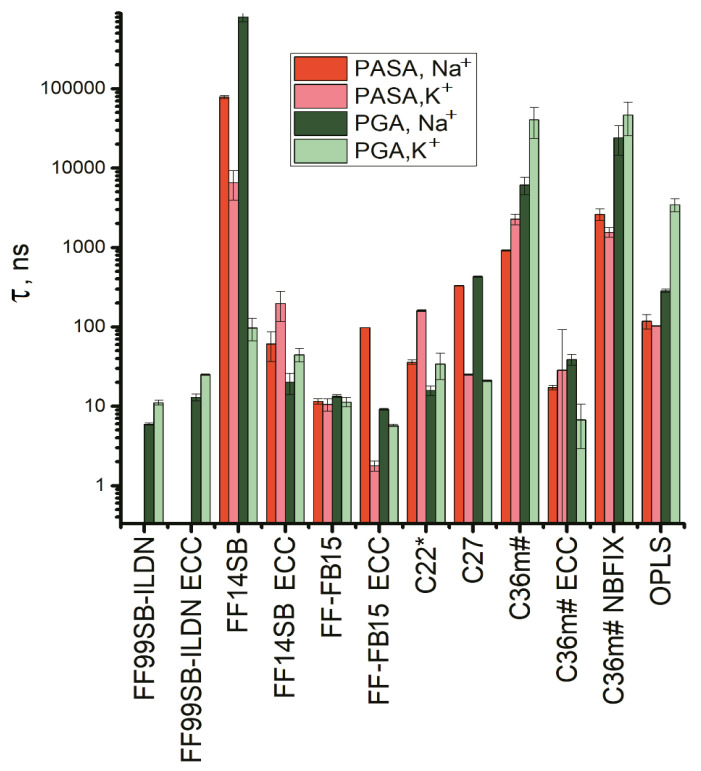
Relaxation times of the dihedral angle Ψ in PASA and PGA molecules with K+ and Na+ counterions. Computed using Equation (2).

The longest relaxation times were obtained for PASA and PGA with Na^+^ for FF14SB, Figure 13. This is explained by stronger stabilization of one of the conformational states (β-conformation) by Na^+^ bridges connecting parts of the molecule into β-sheet structures as discussed in the previous section. As the result, dynamics of the dihedral angles become restricted. In addition, long relaxation times were obtained for C36m# and C36m# NBFIX which can be explained the fact that the C36m# force field was constructed as an improvement to C27 to diminish the probability of the α_R_-helical conformations. As the result, the areas of the accessible dihedral angles decrease and become to the region near PPII conformation (Figures S1 and S2). The same was observed for C36m# with NBFIX. In contrast, the ECC correction leads to a decrease in repulsion between the neighboring carboxyl groups along the chain promoting the lowering of the transition barrier and a consequent decrease of the relaxation time (Figure 13).

## 4. Conclusions

Our data demonstrates that the different force fields yield quantitatively different results. For the α_R_-helical conformations in PASA and PGA, the older force fields tend to overestimate their fraction. This has been also reported for other peptides [57]. C36m (both with and without either of the corrections) and FF14SB (with and without ECC) yielded results that were the most consistent with experimental data [22,23,28,58].

For FF-FB15 and AMBER FF99SB-ILDN, the fraction of α_R_-helical conformations is substantially above the experimental numbers. The addition of the ECC correction to these two force fields makes the situation even more dramatic. The fractions of PPII conformations, for both PASA and PGA with C36m as well as for PASA with C22*, and for PGA with OPLS also strongly exceed those reported in experiments [23,24,29]. Generally, none of the force fields give suitable values for all major backbone conformations.

The fractions of the major backbone conformations did not show significant dependence on counterion parameters with the exception of C27. With K^+^ counterions, C27 gives fractions for the 3.10, PPII and β-conformations for PGA that correspond to the experimental values and the α_R_-helical conformations do not much exceed the experimental numbers (see Table S3). Hence, this force field can be recommended for simulations of PGA but only with K^+^ counterions. It is impossible to make any conclusion for PASA due to lack of experimental data.

For some force fields (FF99SB-ILDN, FF99SB-ILDN ECC, FF-FB15 ECC, C27, and OPLS), long sequences of α_R_-helix conformations appear (both in PASA and PGA). This, however, contradicts experimental data [9,10,11]. In particular, when using FF99SB-ILDN ECC, 15–20% and 40–45% of all monomers can form α_R_- conformational sequences in PASA and PGA chains, respectively.

Direct correlation between increased (decreased) molecular sizes and fractions of stretched (screw) conformations of PASA and PGA monomers was observed in most cases. Opposite behavior was found in the simulations using FF14SB. Regardless of the large number of stretched (β) conformations, their short sequences are connected by Na^+^ bridges into β-sheets which leads to decreased molecular sizes.

The relaxation times for the systems with Na^+^ for some force fields differ from the cases with K^+^ by orders of the magnitude. For PASA with Na^+^, it is observed for the same six force fields as the decreased chain dimensions: FF99SB-ILDN (with and without ECC), FF14SB, FB15-ECC, C27, and C36m#. For PGA with Na^+^, this is the case for FF14SB and C27.

The dihedral angles Φ and Ψ exhibit strongly different relaxation dynamics. For most of the force fields, the Φ angles are strongly restricted. The exception is FF99SB-ILDN, both with and without ECC for PASA. Regarding the relaxation dynamics of the dihedral angle Ψ, Na^+^ counterion specificity leading to an increase in the relaxation time (by one to two orders of magnitude in comparison with K^+^) was observed for C27 and FF14SB. For C27 this is due to the parameterization of Na^+^ giving unrealistically strong interaction between Na^+^ and the carboxyl groups in the PASA and PGA molecules resulting in long-living bridges [14]. FF14SB overestimates the fraction of β-conformational states. This is also explained by the formation of Na^+^ bridges: connecting β-conformational sequences into β-sheet structures. For the other force fields such a behavior is practically absent or even opposite (C22 for PASA, OPLS, and C36m# for PGA). This opposite behavior can be explained by the fact that Na^+^ ions screen the electrostatic repulsion between carboxyl groups, leading to the lowering of the transition barrier.

The ECC corrections added to the C36m#, FF14SB and FF99SB-ILDN, and FF-FB15 force fields have different effects on the results due to differences in the parameterizations of these force fields. When ECC is included in FF99SB-ILDN, the relaxation times for PASA and PGA with both counterions become about doubled. For FF-FB15, the introduction of ECC in the case of PASA with Na^+^ leads to an increase of the relaxation time of about one order of magnitude, but with K^+^ the relaxation time decreases about one order of magnitude. For PGA, the addition of ECC to FF-FB15 has a minimal effect on the relaxation times. In contrast, for C36m# and FF14SB these corrections decrease the dihedral angle relaxation times by two to four orders of magnitude. On the other hand, the NBFIX corrections added to C36m# result in small changes of the monomer structure and dynamic in comparison with native C36m#. This is in agreement with the reported behavior for AMBER force fields being already partially scaled via charge delocalization to some extent [17]. This problem does not seem to affect CHARMM modular force fields [17].

Summarizing, PASA and PGA monomer conformations and dynamics depend on the force field parameterization associated with equilibrium distributions of the rotameric states and the heights of the barriers preventing transitions between these states. They are less dependent on the counterions parameterization with the exception of C27. The default C27 does not perform well with Na^+^ counterions, but with K^+^ counterions it gives good correspondence with experimental data (at least for PGA) unlike other force fields. Due to the lack of experimental data, it is impossible to draw a similar conclusion for PASA.

## Figures and Tables

**Table 1:** Experimental fractions of the different conformations (basins) of aspartic acid (ASA) and glutamic acid (GA) residues in neutral (N) and ionized (I) states. The first column: data for the glycine-glutamic-glycine (Gly-GA-Gly) tripeptide from Ref. [23]; the second column: data from Ref. [24] for the ASA and GA dipeptides; the last two columns: data from Refs. [29,57] for PGA.

Conformation	[23]	[24]				[29]	[57]
	GA	di-ASA		di-GA		PGA	PGA
	N, I	N	I	N	I	I	I
**PPII-helix**	0.54	0.43	0.49	0.59	0.47	0.40	-
**β-sheet**	0.26	0.55	0.46	0.36	0.48	-	0.42
**3.10-helix**	-	-	-	-	-	0.09	-
**α-helix**	0.08	0.02	0.05	0.05	0.05	<0.20	~0.01
**α-helix + β-sheet**	-	-	-	-	-	0.51	-

## Data Availability

Not applicable.

## Supplementary Materials

The following supporting information can be downloaded at: https://www.mdpi.com/article/10.3390/polym14020252/s1. Table S1: List of simulated systems; Table S2: The fractions of PASA monomer conformations; Table S3: The fractions of PGA monomer conformations; Figure S1: PASA Ramachandran plots. Red dots—experimental data; Figure S2: PGA Ramachandran plots. Red dots—experimental data; Figure S3: The integration areas of Ramachandran maps obtained for PASA by using AMBER force fields; Figure S4: Number of the cases of the molecular fragments of different lengths with the same conformations of monomers observed along the PASA simulation trajectory; Figure S5: Number of the cases of the molecular fragments of different lengths with the same conformations of monomers observed along the PGA simulation trajectory; Figure S6: Fractions of different conformations for different force fields; Figure S7: Autocorrelation functions of the time dependence of the end-end distance for all considered systems; Figure S8: Autocorrelation functions of the Ψ and Φ dihedral angles for all considered systems. References [18,21,23,24,29,60,61,62] are cited in supplementary materials.
