# Supplementary material for: Changes in the Local Conformational States Caused by Simple Na+ and K+ Ions in Polyelectrolyte Simulations: Comparison of Seven Force Fields with and without NBFIX and ECC Corrections
Source: Polymers (Basel). 2022 Jan 8;14(2):252. doi: 10.3390/polym14020252 (PMC8779100; doi:10.3390/polym14020252)
Supplement: Supplementary file 1 [file polymers-14-00252-s001.zip › polymers-1531295-supplementary REVISED.pdf]

SUPPLEMENTARY INFORMATION

Changes in The Local Conformational States Caused by Simple Na$^+$ and K$^+$ Ions in Polyelectrolyte Simulations: Comparison of Seven Force Fields with and without NBFIX and ECC Corrections.

Natalia Lukasheva [1,*], Dmitry Tolmachev [1,*], Hector Martinez-Seara [2] and Mikko Karttunen [3,4,5*]

[1] Institute of Macromolecular Compounds, Russian Academy of Sciences, Bolshoy pr. 31, 199004 St. Petersburg, Russia
[2] Institute of Organic Chemistry and Biochemistry, Czech Academy of Sciences, Flemingovo náměstí 542/2, Prague 6 CZ166 10, Czech Republic; hseara@gmail.com
[3] Department of Physics and Astronomy, The University of Western Ontario, 1151 Richmond Street, London, ON N6A 5B7, Canada
[4] The Centre of Advanced Materials and Biomaterials Research, The University of Western Ontario, 1151 Richmond Street, London, ON N6A 5B7, Canada
[5] Department of Chemistry, The University of Western Ontario, 1151 Richmond Street, London, ON N6A 5B7, Canada
* Correspondence: luk@imc.macro.ru (N.L.); dm.tolmachev@yandex.ru (D.T.); mkarttu@uwo.ca (M.K.)

In this study, the following systems were studied:

Table S1. List of simulated systems

№	counterion	force field	polyamino acid	№	counterion	force field	polyamino acid
1	Na$^+$	opls	PGA	25	Na$^+$	opls	PASA
2	K$^+$			26	K$^+$		
3	Na$^+$	amber99sb-ildn		27	Na$^+$	amber99sb-ildn	
4	K$^+$			28	K$^+$		
5	Na$^+$	amber99sb-ildn (ECC)		29	Na$^+$	amber99sb-ildn (ECC)	
6	K$^+$			30	K$^+$		
7	Na$^+$	charmm27		31	Na$^+$	charmm27	
8	K$^+$			32	K$^+$		
9	Na$^+$	charmm22*		33	Na$^+$	charmm22*	
10	K$^+$			34	K$^+$		
11	Na$^+$	charmm36m		35	Na$^+$	charmm36m	
12	K$^+$			36	K$^+$		
13	Na$^+$	charmm36m (NBFIX)		37	Na$^+$	charmm36m (NBFIX)	
14	K$^+$			38	K$^+$		
15	Na$^+$	charmm36m (ECC)		39	Na$^+$	charmm36m (ECC)	
16	K$^+$			40	K$^+$		
17	Na$^+$	amber14sb		41	Na$^+$	amber14sb	
18	K$^+$			42	K$^+$		
19	Na$^+$	amber14sb (ECC)		43	Na$^+$	amber14sb (ECC)	
20	K$^+$			44	K$^+$		
21	Na$^+$	amber fb15		45	Na$^+$	amber fb15	
22	K$^+$			46	K$^+$		
23	Na$^+$	amber fb15 (ECC)		47	Na$^+$	amber fb15 (ECC)	
24	K$^+$			48	K$^+$		

Ramachandran maps for PASA and PGA.

Ramachandran plots presented below in Figures S1 and S2 demonstrate the differences in the conformational space populations for PASA (Figure S1) and PGA (Figure S2) for each tested force field in this study and their comparison with experimental data [21]. Experimental data are shown as red dots on the plots.

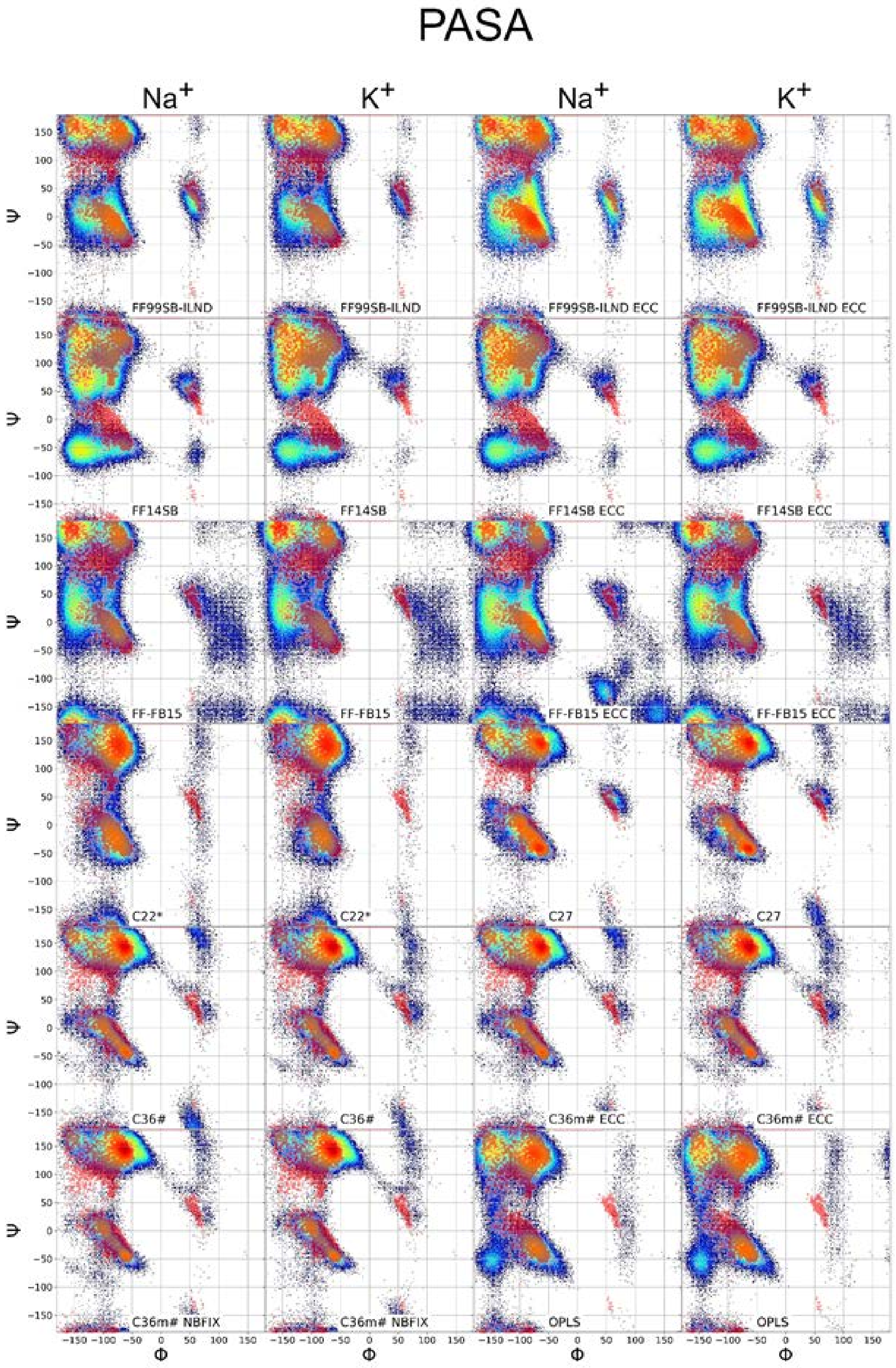

Figure S1. PASA Ramachandran plots. Red dots – experimental data adapted from Ref. [21]

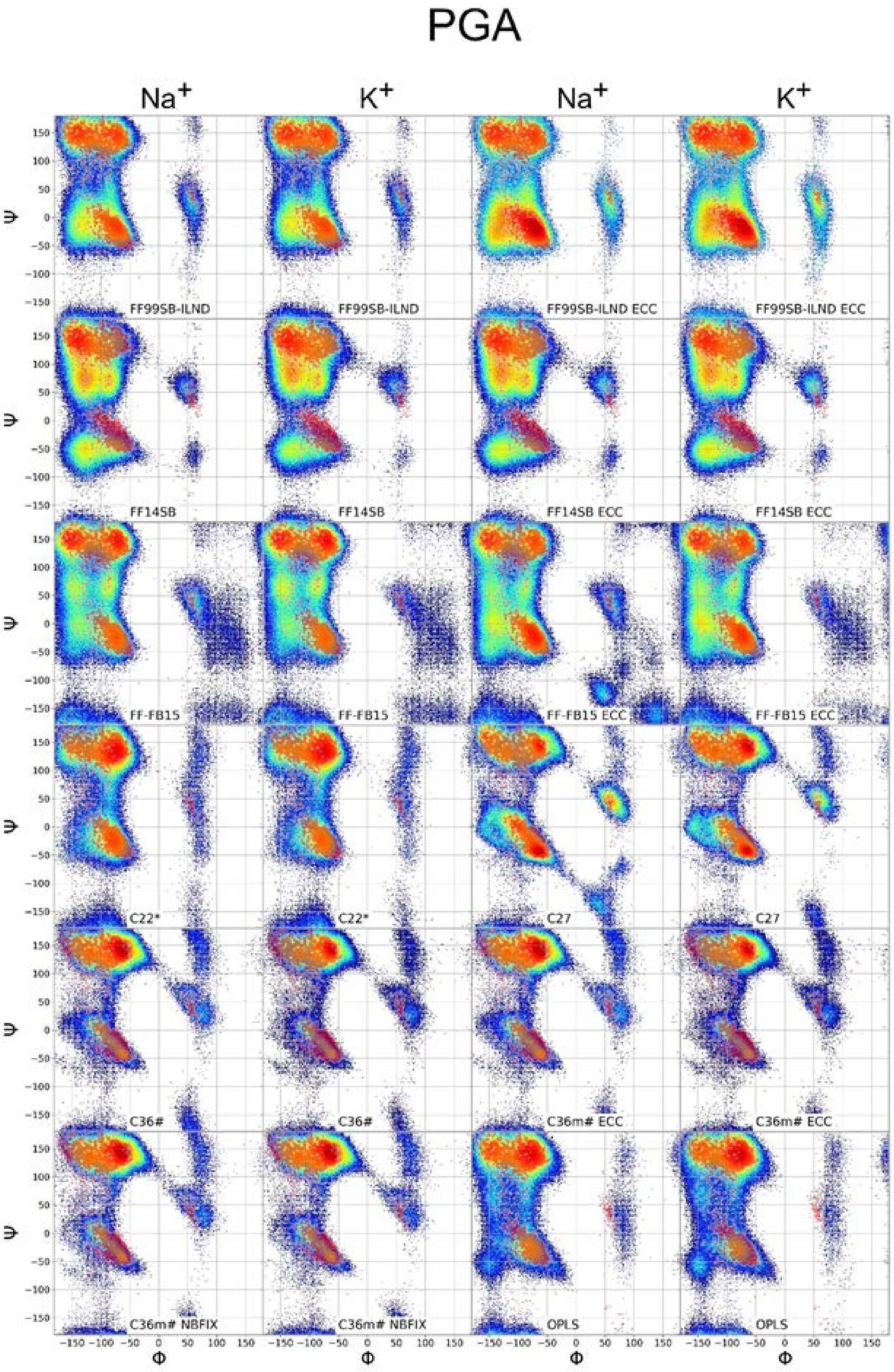

Figure S2. PGA Ramachandran plots. Red dots – experimental data adapted from Ref. [21].

Integration areas of Ramachandran plots allocated for calculation of the conformational fractions.

In Figure S3, the integration areas for PASA Ramachandran plots obtained with AMBER force fields are presented as an example. For PGA, the integration areas are the same.

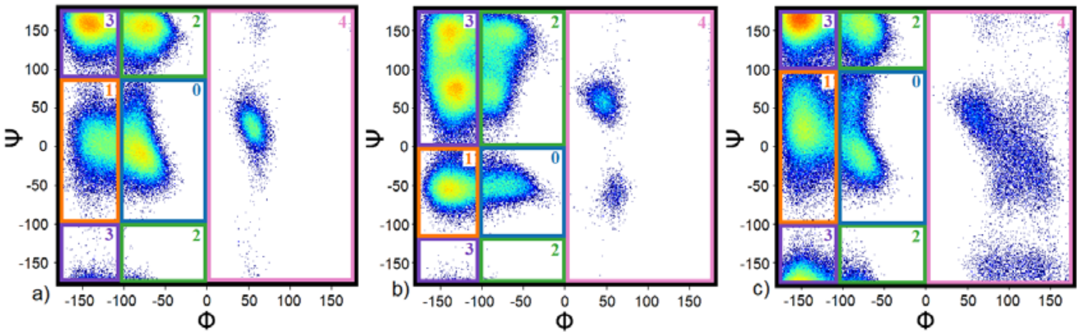

Figure S3. The integration areas of Ramachandran maps obtained for PASA by using AMBER force fields: a) FF99SB-ILDN, b) FF14SB and c) FF-FB15. The areas of dihedral angles related to specific peptide conformations are shown as follows: 0 – α_R helix, 1 – additional screw, 2 – PPII helix, 3 – β sheet, 4 – φ > 0° (mostly left handed α helix).

The calculated fractions of PASA and PGA monomer conformations are presented below in Tables S2 and S3, respectively.

Table S2. The fractions of PASA monomer conformations. The values in agreement with experiments are indicated in green color. Additional screw conformations are named add. scr. Force fields for which 3.10 were estimated are indicated in blue color.

	K^+				Na^+			
	α	3.10, add. scr.	PPII	β	α	3.10, add.scr.	PPII	β
Experiment [24]	0.05	---------	0.49	0.46	0.05	--------	0.49	0.46
FF99sb-ildn	0.13±0.02	0.15±0.02	0.30±0.02	0.41±0.02	0.24±0.03	0.15±0.03	0.25±0.02	0.33±0.04
FF99sb-ildn ECC	0.31±0.03	0.21±0.02	0.18±0.02	0.23±0.02	0.34±0.03	0.16±0.04	0.19±0.03	0.27±0.03
FF14sb	0.04±0.01	0.09±0.02	0.25±0.02	0.61±0.02	0.06±0.02	0.15±0.03	0.24±0.02	0.52±0.03
FF14sb ECC	0.03±0.01	0.10±0.02	0.24±0.01	0.62±0.02	0.04±0.01	0.10±0.02	0.24±0.02	0.62±0.02
FF fb15	0.10±0.02	0.20±0.03	0.14±0.02	0.51±0.06	0.13±0.04	0.20±0.03	0.14±0.02	0.47±0.06
FF fb15 ECC	0.19±0.04	0.26±0.02	0.10±0.0	0.42±0.04	0.27±0.05	0.26±0.04	0.12±0.03	0.27±0.04
C22*	0.12±0.02	0.07±0.01	0.72±0.03	0.09±0.01	0.18±0.03	0.08±0.01	0.64±0.05	0.10±0.01
C27	0.20±0.07	0.11±0.02	0.54±0.06	0.13±0.02	0.24±0.03	0.16±0.02	0.37±0.08	0.21±0.05
C36m#	0.06±0.01	0.06±0.02	0.77±0.03	0.08±0.01	0.10±0.01	0.09±0.02	0.63±0.05	0.15±0.03
C36m# ECC	0.07±0.01	0.06±0.01	0.76±0.03	0.08±0.01	0.06±0.02	0.06±0.01	0.77±0.03	0.09±0.01
C36m# NBFIX	0.06±0.01	0.06±0.02	0.77±0.03	0.08±0.01	0.06±0.01	0.05±0.02	0.79±0.02	0.08±0.01
OPLS	0.18±0.09	0.05±0.01	0.42±0.05	0.34±0.04	0.15±0.07	0.05±0.01	0.52±0.05	0.28±0.03

Table S3. The fractions of PGA monomer conformations. The values in agreement with experiments are indicated in green color. Additional screw conformations are named add. scr. Force fields for which 3.10 were estimated are indicated in blue color

		K^+				Na^+			
		α	3.10, add. scr.	PPII	β	α	3.10, add. scr.	PPII	β
Experiment	[29]	0.05	0.09	0.4	0.26	0.05	0.09	0.4	0.26
	[23]	0.08		0.54	0.48	0.08		0.54	0.48
FF99sb-ildn		0.25±0.03	0.19±0.02	0.25±0.03	0.30±0.03	0.27±0.05	0.19±0.02	0.24±0.04	0.29±0.03
FF99sb-ildn ECC		0.39±0.06	0.20±0.02	0.17±0.04	0.20±0.04	0.44±0.04	0.23±0.03	0.14±0.02	0.17±0.02
FF14sb		0.04±0.01	0.08±0.02	0.29±0.02	0.58±0.02	0.07±0.02	0.11±0.01	0.23±0.02	0.59±0.02
FF14sb ECC		0.05±0.01	0.11±0.03	0.28±0.02	0.54±0.03	0.05±0.01	0.09±0.02	0.28±0.02	0.56±0.03
FF fb15		0.22±0.05	0.17±0.03	0.32±0.05	0.29±0.02	0.24±0.05	0.16±0.03	0.28±0.06	0.30±0.02
FF fb15 ECC		0.31±0.07	0.23±0.04	0.18±0.03	0.26±0.04	0.37±0.08	0.21±0.03	0.16±0.04	0.24±0.04
C22*		0.12±0.02	0.09±0.01	0.61±0.04	0.17±0.01	0.16±0.03	0.11±0.02	0.55±0.04	0.16±0.01
C27		0.18±0.08	0.11±0.02	0.44±0.06	0.22±0.03	0.28±0.03	0.19±0.02	0.24±0.04	0.19±0.05
C36m#		0.02±0.01	0.03±0.01	0.79±0.02	0.13±0.01	0.03±0.01	0.04±0.01	0.77±0.04	0.13±0.01
C36m# ECC		0.04±0.01	0.05±0.01	0.74±0.03	0.14±0.01	0.04±0.01	0.05±0.01	0.79±0.03	0.13±0.01
C36m# NBFIX		0.02±0.01	0.03±0.01	0.80±0.03	0.13±0.01	0.03±0.01	0.04±0.01	0.75±0.03	0.13±0.01
OPLS		0.06±0.02	0.02±0.00	0.72±0.03	0.19±0.01	0.09±0.02	0.02±0.01	0.65±0.03	0.23±0.01

Procedure for estimation of the fractions of carboxyl groups involved in the Na^+ bridges formation.

The coordination of a counterion with at least of two oxygens belonging to different carboxyl groups was considered as a counterion bridge. The fraction of bridges was calculated as the difference between the average number of carboxyl groups coordinated with an ion (divided by the number of carboxyl groups) (ν_{carb}) and the fraction of adsorbed ions (ν_{ads})

$$\nu_{rb} = \nu_{carb} - \nu_{ads} \qquad \text{(S1)}$$

The average number of carboxyl groups coordinated with an ion was estimated the same way as the fraction of adsorbed ions.

Molecular fragments with different lengths consisting of monomers with the same conformation ("regular regions").

The number of the appearances of the molecular fragments of different lengths with the same conformations of monomers along the trajectory were estimated and presented in Figures S4 and S5.

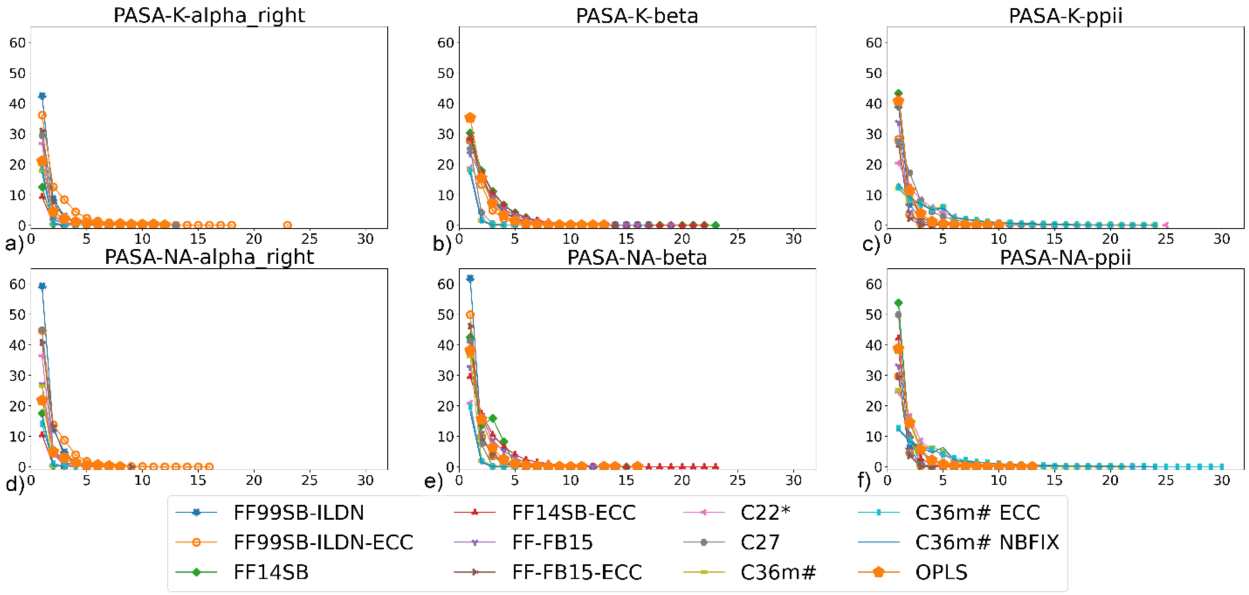

Figure S4. Number of the cases of the molecular fragments of different lengths with the same conformations of monomers observed along the PASA simulation trajectory. a), b), c) simulation with K^+ counterions; d), e), f) simulation with Na^+ counterions. a), d) right-handed α helix; b), e) β-sheet; c), f) PPII helix.

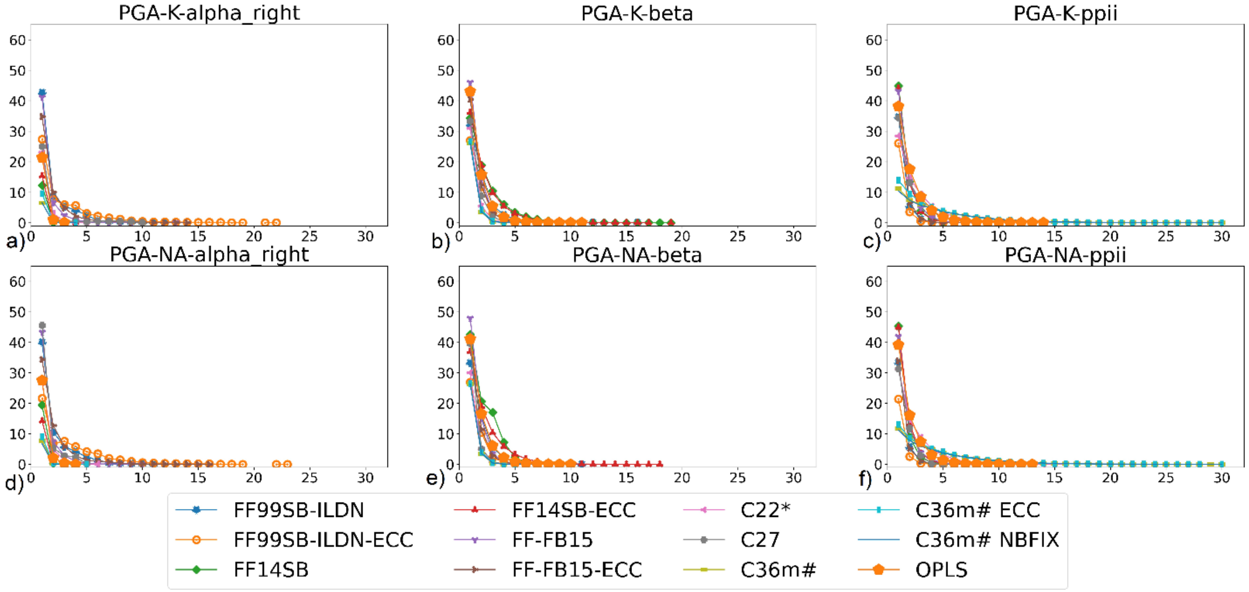

Figure S5 Number of the cases of the molecular fragments of different lengths with the same conformations of monomers observed along the PGA simulation trajectory. a), b), c) simulation with K^+ counter ions; d), e), f) simulation with Na^+ counter ions. a), d) right-handed α-helix; b), e) β–sheet; c), f) PPII helix.

Fractions of different conformations in PASA and PGA molecules.

Calculated from the Ramachandran maps, the fractions of the different conformations in PASA and PGA molecules with Na^+ and K^+ counterions obtained for the different force fields are presented for clarity in graphs in Figure S6. This gives the additional information to Figure 9 in the main text.

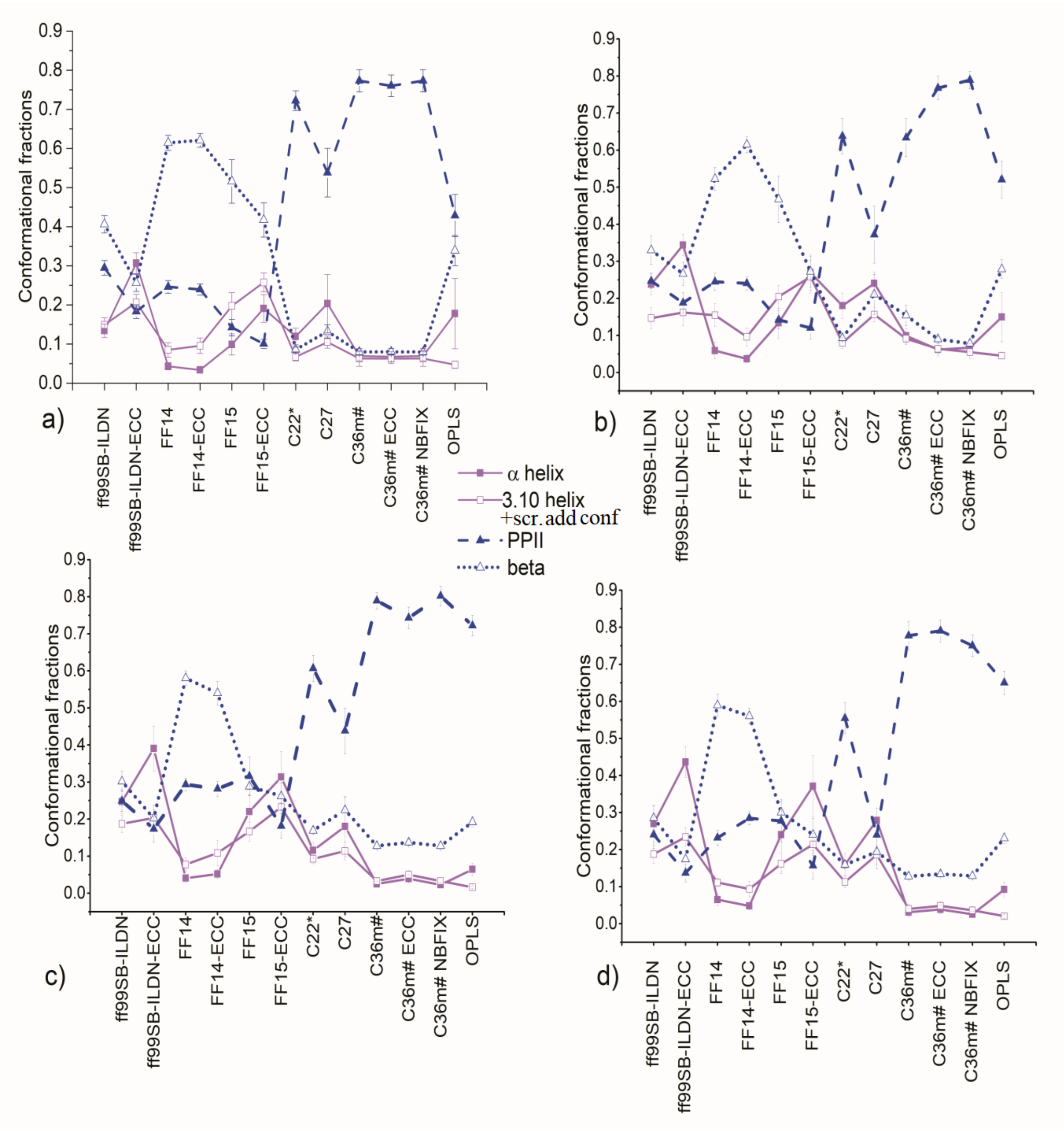

Figure S6. Fractions of different conformations in a), b) PASA and c), d) PGA molecules; with a),c) K^+ and b), d) Na^+ counterions for different force fields.

Autocorrelation functions

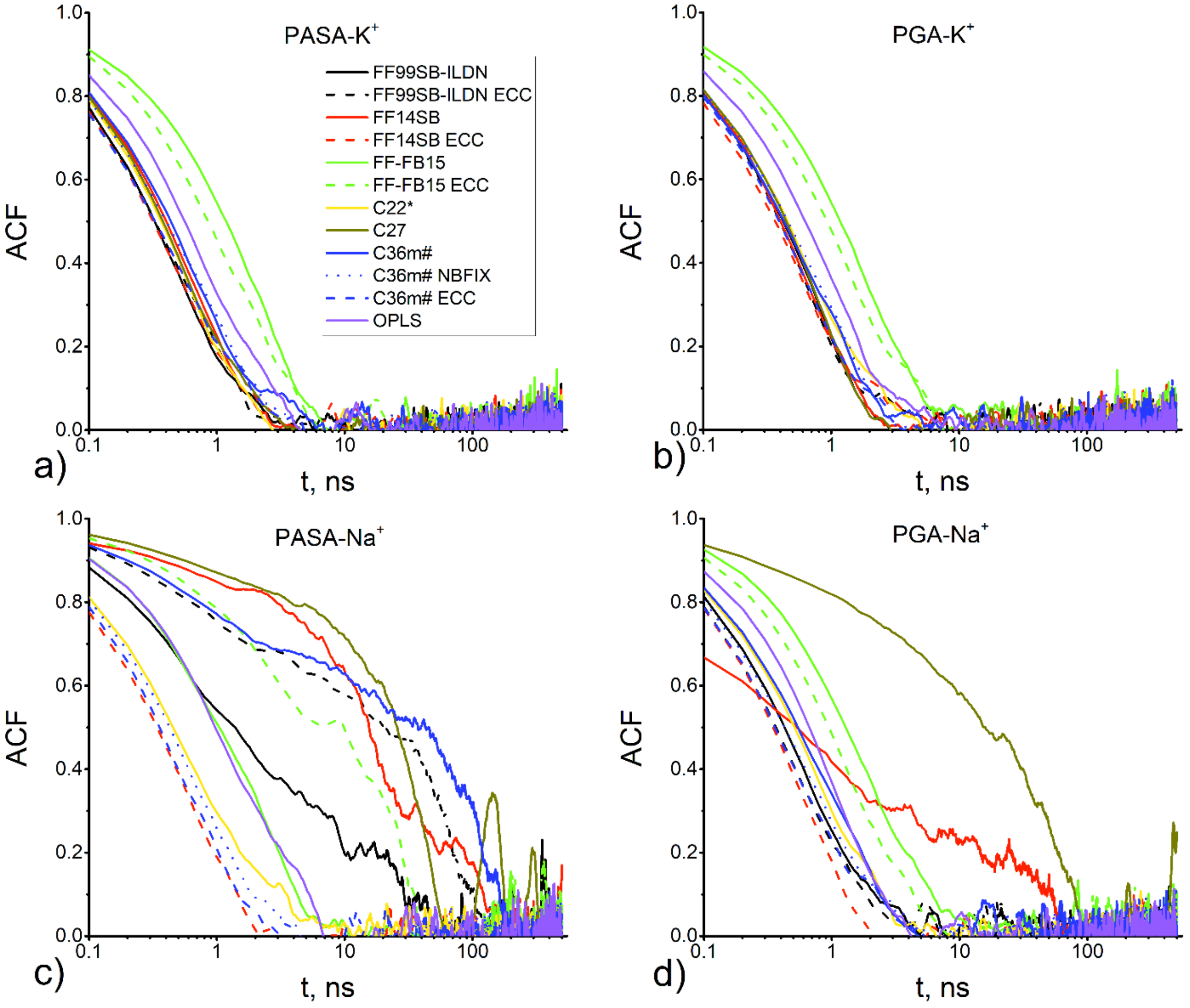

Figure S7. Autocorrelation functions of the time dependence of the end-end distance for all considered systems.

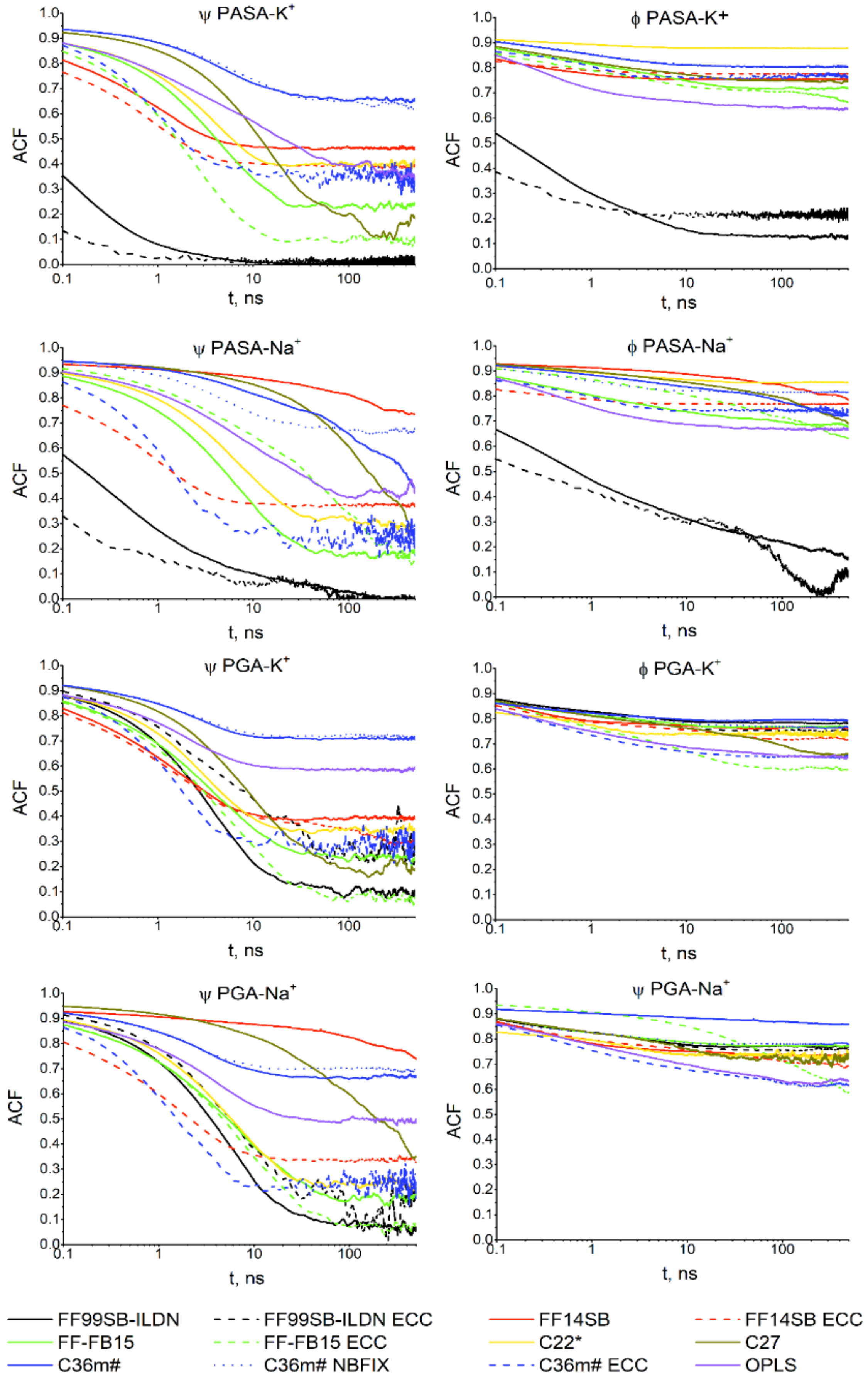

Figure S8. Autocorrelation functions of the Ψ and Φ dihedral angles for all considered systems.

Φ, Ψ peptidic dihedral angle autocorrelation functions

The Φ, Ψ peptidic dihedral angle autocorrelation functions in Figure S8 reflect the dynamics of the transitions inside the allowed conformational areas and between them (Figures S1 and S2). The dihedral autocorrelation functions for PASA and PGA decay from <0.1 ns to hundreds or even thousands of nanoseconds due to the dynamical processes involving backbone and side-chain local motions, backbone segmental dynamics, long-range correlated dynamics, and other processes [60–62].

The dynamics of the Φ angles is restricted in most cases and the autocorrelation function behavior demonstrates weak dependence on the force field and counterion type (Figure S8). The regions in the Ramachandran plots for Φ (see Figures S1 and S2) are narrow and only restricted fluctuations near the strongly stabilized PPII or α-helical conformations are possible. It affects the autocorrelation functions of Φ angles and its relaxation times (see Figure S8b) The exception is FB99SB-ILDN (with and without ECC) for PASA only. Probably this is due to the improvements for the side-chain torsion potentials for aspartic acid to achieve better than FF99SB agreement with experiment [18]. FF99SB-ILDN gives for PASA a significantly wider distribution for angle Φ and quicker decay of the autocorrelation curve.
